# Peer Assessment Enhances Student Learning: The Results of a Matched Randomized Crossover Experiment in a College Statistics Class

**DOI:** 10.1371/journal.pone.0143177

**Published:** 2015-12-18

**Authors:** Dennis L. Sun, Naftali Harris, Guenther Walther, Michael Baiocchi

**Affiliations:** 1 Department of Statistics, California Polytechnic State University, San Luis Obispo, CA, 93407, United States of America; 2 Department of Statistics, Stanford University, Stanford, CA, 94305, United States of America; 3 Prevention Research Center, Stanford School of Medicine, Stanford, CA, 94305, United States of America; University of Westminster, UNITED KINGDOM

## Abstract

Feedback has a powerful influence on learning, but it is also expensive to provide. In large classes it may even be impossible for instructors to provide individualized feedback. Peer assessment is one way to provide personalized feedback that scales to large classes. Besides these obvious logistical benefits, it has been conjectured that students also learn from the practice of peer assessment. However, this has never been conclusively demonstrated. Using an online educational platform that we developed, we conducted an in-class matched-set, randomized crossover experiment with high power to detect small effects. We establish that peer assessment causes a small but significant gain in student achievement. Our study also demonstrates the potential of web-based platforms to facilitate the design of high-quality experiments to identify small effects that were previously not detectable.

## Introduction

Feedback is one of the single most important factors influencing student learning [1, 2]. However, it is often not possible to provide feedback that is both detailed and prompt, thus limiting its effectiveness in practice [3]. In large college classes and massively open online courses (MOOCs), providing personalized feedback to students is especially challenging.

While automated feedback can be an adequate substitute in some cases [4], many concepts and skills are still challenging for a machine to evaluate. Consider the following question about the interpretation of a p-value, an important concept in introductory statistics.

Josh flips a coin 100 times. The coin comes up heads 60 times. He calculates the p-value to be about 2% for testing the null hypothesis that the coin is fair. Explain what this 2% means in the context of this problem.

The correct answer is that the 2% represents the probability of observing a result at least this large if the coin were fair. However, a common misconception among students is that it represents the probability the coin is fair. Even with state-of-the-art semantic parsing, machines cannot accurately discriminate incorrect answers from correct ones [5]. On the other hand, a human who understands the concept would have little difficulty distinguishing the two.

Thus, the problem of providing feedback falls into the large class of tasks that are relatively easy for a human but challenging for a machine. Such tasks are fertile ground for crowdsourcing, which has been applied to otherwise intractable problems with surprising success [6, 7]. Feedback can be “crowdsourced” by having students grade one another, a practice known as *peer assessment*. Peer assessment provides as many graders as students, enabling more timely and thorough feedback [8]. It has already made personalized feedback feasible in a number of settings—most notably MOOCs—where it otherwise would be impossible [9].

Instructors often have two main concerns about peer assessment. The first is whether students can be trusted to grade accurately. This question has been extensively studied in the literature, and the consensus is that peer grades are at least comparable to instructor grades [10, 11]. The second is logistics; peer assessment is logistically complicated if students have to exchange papers in person. However, web-based tools have largely solved this problem. Most modern learning management systems (LMS) come with a built-in peer assessment tool that automatically distributes student responses to peer graders.

Therefore, peer assessment is a workable solution to the problem of feedback; it reduces the burden to the instructors with minimal sacrifice to quality. On top of this, it has been conjectured that students also learn in the process of providing feedback. If true, then peer assessment may be more than just a useful tool to manage large classes; it can be a pedagogical tool that is both effective and inexpensive [8, 12, 13].

This claim was perhaps most visibly advanced in the U.S. Supreme Court case *Owasso v*. *Falvo* (2002) [14]. Although the case was primarily concerned with whether peer assessment violated students’ privacy, Justice Anthony Kennedy praised peer assessment in his majority opinion, saying

Correcting a classmate's work can be as much a part of the assignment as taking the test itself. It is a way to teach material again in a new context, and it helps show students how to assist and respect fellow pupils [11].

However, to date, there has been scant empirical evidence for this claim. The evidence is based mostly on surveys of student and teacher perceptions [8, 15]. Only a few studies have attempted to quantify the effect on an objective criterion such as achievement, but most have been correlational studies. A representative study in this latter category is [16], which examined whether peer assessment improved students’ writing abilities. However, the study lacked a control group, so it is not possible to know whether students improved any more with peer assessment than they would have otherwise. Furthermore, the study measured achievement using the students’ own peer grades, rather than an objective measure (e.g., scores given by a third-party observer who was blinded to the treatment). To our knowledge, only one randomized experiment has ever been conducted to measure the effect of peer assessment on achievement, but the study lacked statistical power to reach a conclusion either way [17]. This gap in the literature has been noted by several researchers, who have suggested this as an important direction of future research [17, 18].

## Materials and Methods

We were interested in whether peer assessment could aid conceptual understanding and problem solving, two skills that are especially relevant in science, technology, engineering, and mathematics (STEM) classes. We conducted a randomized controlled trial (RCT) in a large introductory statistics class, using a crossover design to enhance precision. Written, informed consent was obtained from all participants, and the study was approved by the Stanford University Institutional Review Board. The ten-week course was divided into an introductory unit and four main units. The introductory unit was excluded from the study so that students had time to become acclimated to peer assessment. Each student was then assigned to participate in peer assessment for exactly two of the main units and to the control group for the other two. In all, there were four treatment arms, each one receiving the peer assessment treatment (T) and control (C) in a different order over the four main units (TCTC, CTCT, TCCT, and CTTC).

**Fig 1:**
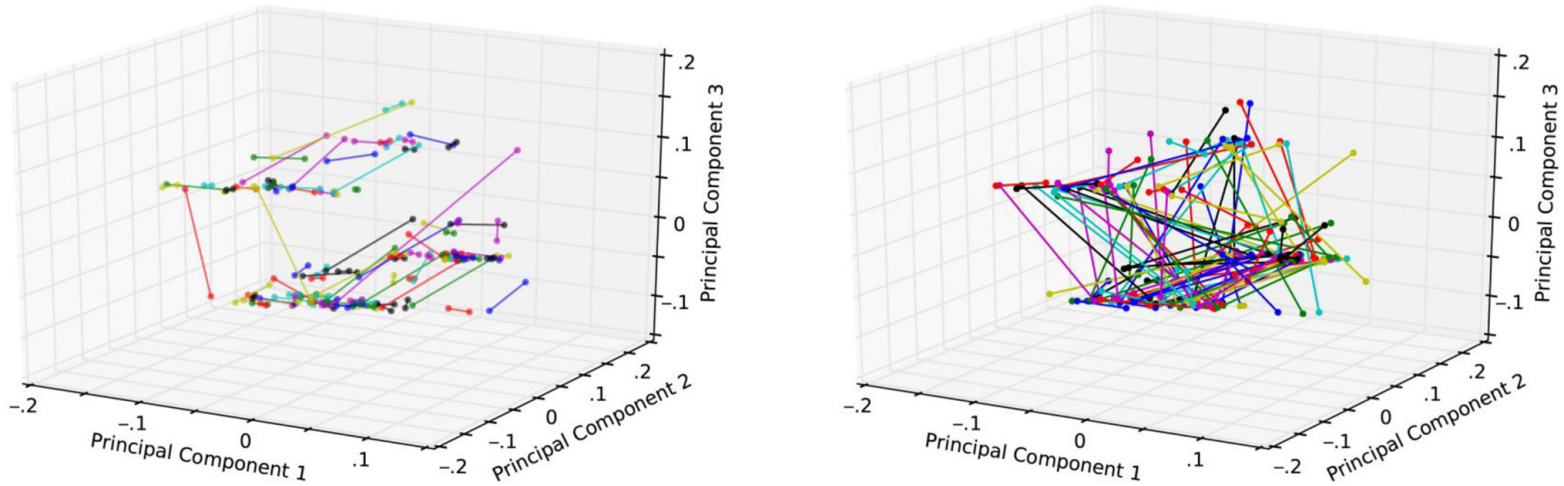
Two plots showing the effect of the matched pairs randomization design (1A) as compared with complete randomization (1B). Each point represents a student’s covariate information, and each connecting edge indicates that those students have been assigned to opposite treatment groups. The edges in the matched pairs design are much shorter than under complete randomization, confirming that matching produces more similar randomizations.

This crossover design controls for all differences between students, since each student participates both as a treatment and a control subject at different times in the course. This eliminates what is arguably the largest source of variability in educational studies: variation between students. To further ensure against baseline differences between the treatment and control groups, we augmented this design with matched-pairs randomization. Students were matched based on covariate information (e.g., class year, previous statistics experience), and the students within each pair were assigned to complementary treatments in all four of the main units (i.e., TCTC vs. CTCT). Fig 1 shows the result of the matched pairs design, alongside a random pairing for comparison. The resulting covariate balance for the actual randomization is provided in (S1 File), showing that the matched-pairs design produced indistinguishable groups.

The treatment was defined as follows: in weeks that students were required to participate in peer assessment, they provided scores and comments on the homework responses of three peers. In turn, they received feedback on their homework from three peers. All homework responses and peer assessments were submitted through an online platform, and all responses and reviews were anonymized before distribution to ensure privacy. Students in the control group also submitted their homework responses online but did not participate in peer assessment and had their homework graded by instructors. In order to control for the possible effect of feedback timing, feedback was delivered to the two groups simultaneously. Also, the students in the control group were provided the same solution key as the one provided to the peer graders. For more details about the specific implementation of peer assessment, please refer to (S1 File).

To measure achievement, students completed a quiz after each unit that measured the short-term effect of peer assessment. The students also took a comprehensive final exam that measured longer-term learning. These assessments consisted entirely of free-response questions that required explanations or calculations. The instructors, who were blinded to the treatment groups of the students, graded all assessments to ensure consistency for the purposes of the study.

Finally, the study was fully replicated in a different academic term with a different instructor. The same homework questions were used in the two terms, but different exam questions were used. In all, 148 students participated in the study during the first term (autumn) and 239 students during the second (winter). Because the crossover design should eliminate any student or instructor effects, we pooled the data from the two terms to obtain a single sample of 387 students. However, in the analyses, we excluded any students who failed to comply with the peer assessment treatment or to complete the assessments. This left us with 299 students in the analysis of the unit quiz scores and 320 students in the analysis of the final exam scores. Although excluding non-compliers can sometimes bias the treatment effect, our crossover design ensures that non-compliers are excluded from both the treatment and control groups. As a result, we obtain an unbiased estimate of the treatment effect on the subpopulation of students who would be affected by the peer assessment intervention. A further discussion of this and the definition of compliance can be found in (S1 File).

## Results

**Fig 2:**
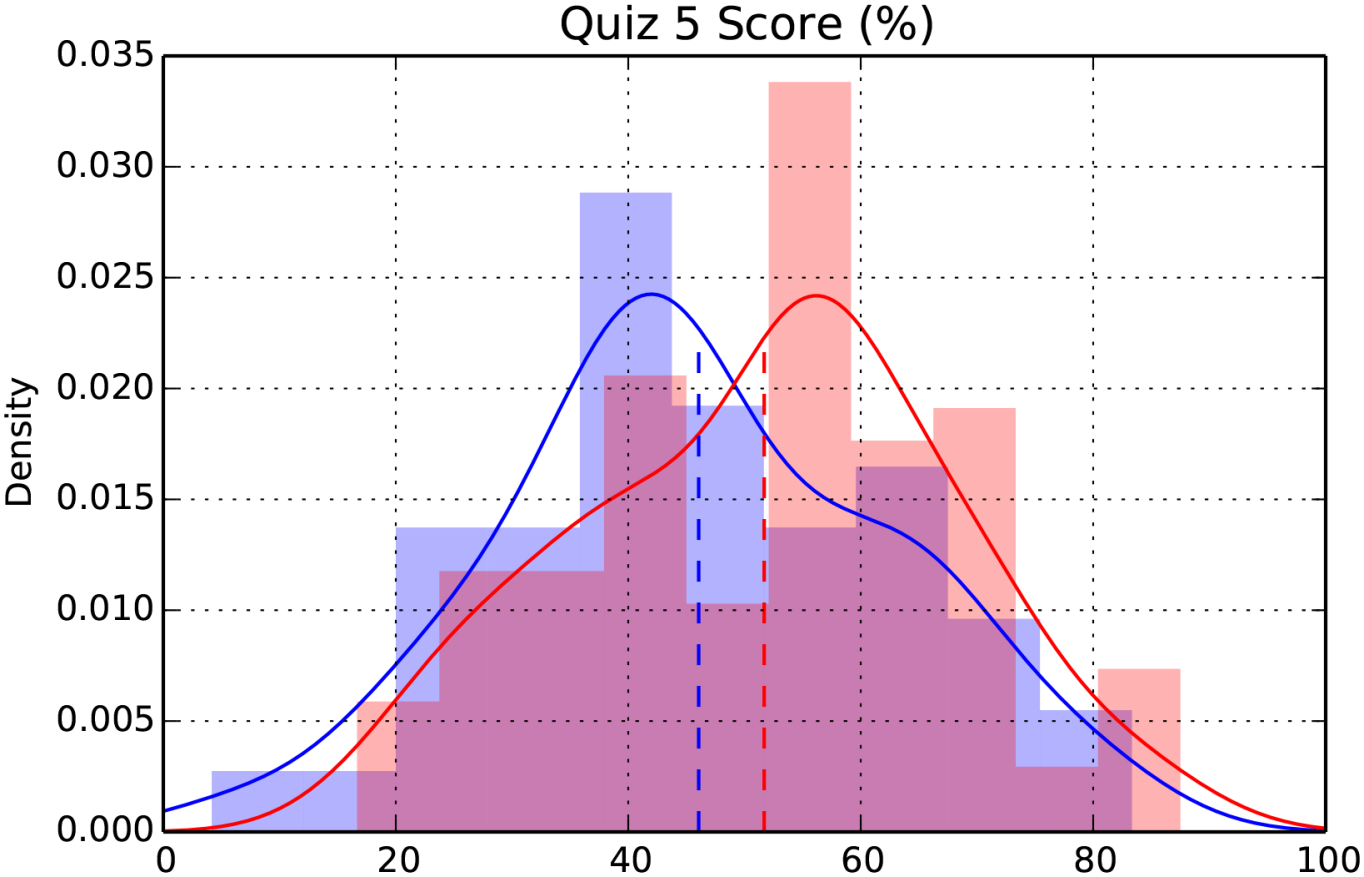
Distribution of scores for the control (blue) and treatment (red) groups on quiz 5 in the winter quarter. The dashed vertical lines designate the means. (The difference in means on this quiz was 5.9.) Similar plots for all of the quizzes and final exam may be found in (S1 File).

The students who participated in peer assessment during a given unit performed significantly better on the unit quizzes (Cohen's d = .115, t(298) = 2.92, p = .002), as compared with students who did not. Students participating in peer assessment also did better on the corresponding questions on the final exam (d = .122, t(319) = 3.03, p = .001), which suggests that the benefits of peer assessment persist over time. These results are summarized in Table 1. In the context of our course, where the standard deviations of exam scores ranged from 15 to 25 percentage points, an effect size of .122 would translate to a 2 to 3 percentage point increase in the average exam score. Fig 2, which depicts the actual distribution of scores for one of the unit quizzes, shows that a modest increase in average score can be practically important.

**Table 1:** The effect sizes of peer assessment in the short term and long term. (Standard errors are shown in parentheses.)

Type of Effect	Effect Size
Short Term (as measured by unit quizzes)	0.115 (0.04)
Long Term (as measured by final exam)	0.122 (0.04)

To understand the magnitude of this effect size, we also calculated the “effect sizes” of well-known achievement gaps: between males and females, between underrepresented minorities and others, between students with more math background and students with less, etc. These achievement gaps are reported in Table 2. We estimated the gap twice, once at the beginning of the course (using quiz 1 scores, which was administered prior to randomization) and again at the end of the course (using final exam scores). We see that the effect size of peer assessment, which is .122, represents about 40% of the gender achievement gap and about 20% of the racial achievement gap, which are persistent challenges in college science classes [19, 20]. By comparing the gaps before and after the course, we see also that the course tended to reduce achievement gaps, although we do not have enough evidence to attribute this to the peer assessment intervention.

**Table 2:** Achievement gaps in our population of students, reported as an effect size. We show the gap before and after the course. (Standard errors are shown in parentheses.) The “before” numbers were calculated using scores on a pre-quiz administered prior to the randomization. The “after” numbers were calculated using scores on the final exam.

Achievement Gap	Difference before Course	Difference after Course
Gender achievement gap (1 = male)	0.32 (0.12)	0.13 (0.12)
Racial achievement gap (1 = underrepresented minority)	−.61 (0.14)	−.42 (0.13)
Statistics background (1 = passed AP stats)	0.54 (0.11)	0.59 (0.12)
Math background (1 = course beyond calculus)	0.68 (0.10)	0.54 (0.11)
Class year (1 = upperclassman)	0.22 (0.12)	0.07 (0.11)

We also surveyed students on their perception of the benefit of peer assessment on a scale from 1 to 5, with 1 indicating “not helpful at all" and 5 indicating “extremely helpful". Although the median student reported finding peer assessment only “somewhat helpful," there was virtually zero correlation (r = .01, p = .94) between a student's perception of the benefit and the estimated benefit. This lends credence to our concern that surveys may not be the best measure of student learning. A further analysis of student reaction to peer assessment can be found in (S1 File).

## Discussion

This study has established that peer assessment produces concrete gains in student achievement, above and beyond the effect of receiving feedback. Thus, peer assessment is unique among educational interventions in that the usual cost-benefit tradeoff seems not to apply: it saves instructors time, while also benefiting students. This suggests that peer assessment should not be limited to MOOCs and large classes where it is the only option, but that it has a place even in smaller settings where it is not strictly needed.

This study is also a demonstration of the role that web-based platforms, such as learning management systems and MOOCs, can play in education research. The ability to personalize an individual’s experience using such platforms is easier than in traditional formats. This makes individual-level randomizations, the core of high quality RCTs, much easier. While we have focused on peer assessment specifically, a similar study design could be used to investigate other questions as well. The transformational impact of web-based educational tools may be their ability to facilitate experiments in the classroom, enabling us to obtain unprecedented insight into the learning process.

## Supporting Information

S1 File Supplementary Information for *Peer assessment enhances student learning*. (PDF)
